# Smart Hydrogel Bilayers Prepared by Irradiation

**DOI:** 10.3390/polym13111753

**Published:** 2021-05-27

**Authors:** Weixian Huo, Heng An, Shuquan Chang, Shengsheng Yang, Yin Huang, Xiaohong Zhang, Xiaodan Hu, Haiqian Zhang

**Affiliations:** 1 Key Laboratory of Nuclear Technology Application and Radiation Protection in Astronautics, College of Material Science and Technology, Nanjing University of Aeronautics and Astronautics, Nanjing 210016, China; huo_weixian@163.com (W.H.); sin0009@163.com (Y.H.); Xiaohongzhang@nuaa.edu.cn (X.Z.); huxiaodan@nuaa.edu.cn (X.H.); Zhanghq@nuaa.edu.cn (H.Z.); 2 Science and Technology on Vacuum Technology and Physics Laboratory, Lanzhou Institute of Physics, Lanzhou 730000, China; ahllbl@126.com (H.A.); 2syang@sina.com (S.Y.)

**Keywords:** composite hydrogel, irradiation, bilayer, temperature-sensitive

## Abstract

Environment-responsive hydrogel actuators have attracted tremendous attention due to their intriguing properties. Gamma radiation has been considered as a green cross-linking process for hydrogel synthesis, as toxic cross-linking agents and initiators were not required. In this work, chitosan/agar/P(N-isopropyl acrylamide-co-acrylamide) (CS/agar/P(NIPAM-co-AM)) and CS/agar/Montmorillonite (MMT)/PNIPAM temperature-sensitive hydrogel bilayers were synthesized via gamma radiation at room temperature. The mechanical properties and temperature sensitivity of hydrogels under different agar content and irradiation doses were explored. The enhancement of the mechanical properties of the composite hydrogel can be attributed to the presence of agar and MMT. Due to the different temperature sensitivities provided by the two layers of hydrogel, they can move autonomously and act as a flexible gripper as the temperature changes. Thanks to the antibacterial properties of the hydrogel, their storage time and service life may be improved. The as prepared hydrogel bilayers have potential applications in control devices, soft robots, artificial muscles and other fields.

## 1. Introduction

Biological organisms can change their shape when environmental conditions stimulate them. Inspired by this, soft actuators [1,2] have drawn the attention of domestic and foreign scholars. In recent years, compared to electric or hydraulic hard actuators, soft actuators have become popular due to their flexibility and adaptability. Bajaj and colleagues reported an underactuated design of a soft hand exoskeleton for grasping and lifting objects [3]. Xiang and co-workers have synthesized a smart Janus, which can reversely grab objects rapidly under high humidity conditions. Moreover, the Janus was constructed by asymmetric polymer brushes on polydimethylsiloxane as a substrate through surface-initiated atom transfer radical polymerization [4]. The soft actuator can be used in the soft hand exoskeleton and for grabbing objects, etc.

Smart hydrogels can observably change their volume, color, and other properties under the stimulation of the external environment, such as temperature, pH, ionic strength, light, electric field, magnetic field, etc. In addition, hydrogels usually have well-defined structures that can be modified to yield tailorable functionality [5]. Therefore, the use of smart hydrogel as a soft actuator is a research hotspot recently. Widely-studied hydrogel actuators are made of double-layer hydrogels. Hyojin and co-workers synthesized a hydrogel ink composed of acrylamide, N-isopropylacrylamide and sugar. With the use of 4D printing, they created a printed structure that mimicked the shape and petal movements of a bellflower [6]. Zhang and co-workers constructed a monolithic robust actuator of a binary cooperative Janus, which was synthesized by interfacial polymerization of immiscible hydrophilic and hydrophobic vinyl monomer solutions, and applied it to leakage detection [7]. Gao and co-workers prepared poly (NIPAM-co-DMAPMA)/clay hydrogel bilayers, which can jump with a change in temperature [8]. Chen and co-workers designed a novel thermal-/NIR-responsive double network structure of hydrogel bilayers [9]. Many researchers developed soft actuators made by smart hydrogels, which will provide potential benefits for soft robots [10,11,12], artificial muscles [13,14,15], and drug delivery [16]. However, most of the existing studies used chemical reagents for cross-linking, and the preparation of hydrogel bilayers by gamma ray is rarely reported. Compared with the traditional chemical method, the preparation of hydrogel actuators by gamma radiation is simple and environmentally friendly. In the radiation assistant preparation method, there is no need to add a cross-linking agent or initiator, and the introduction and residue of toxic substances are avoided to meet the requirements of green chemistry [17,18]. Furthermore, much of the literature has indicated that bacteria can degrade hydrogels and affect their performances [19,20]. Therefore, the preparation of hydrogel actuators with bacteriostatic action is an urgent issue.

Herein, we prepared a temperature-sensitive hydrogel actuator containing chitosan by gamma radiation. Previous studies have reported that hydrogels with antibacterial properties can prolong the life of substances, increasing the storage time and service life of the hydrogel actuator [21,22,23]. Gamma ray sterilization is a safe and effective method [24,25,26]. The use of gamma ray cross-linking hydrogels does not require secondary sterilization. Additionally, it has antibacterial properties due to chitosan (CS); it can realize the integration of sterilization and bacteriostasis. N-isopropylacrylamide (NIPAM) is one of the most typical materials among the temperature-sensitive hydrogels with volume phase transition temperature (VPTT). When the temperature is lower than the VPTT, the hydrophilic groups in the molecular chain can form hydrogen bonds with water molecules. The hydrogel is hydrophilic and can swell in water. When the volume phase transition occurs, the hydrogel exhibits hydrophobic properties with hydrogen bond breaks, and the volume shrinks with the change in the hydrogel structure [27,28,29,30]. This property of temperature-responsive volume changing plays an essential role in the hydrogel bilayer. Therefore, NIPAM was added as the temperature-sensitive body of the smart hydrogel. In addition, agar can improve the mechanical properties of hydrogels [31], and chitosan can provide antibacterial properties. Different amounts of acrylamide (AM) were added to the two layers of gel to make the volume phase transition temperature of the two layers of hydrogel different. The water molecules can produce free radicals under gamma radiation, and the free radicals can react with polymer chains to cross-link the hydrogel. In this way, we have successfully prepared hydrogel bilayers, which have the properties of storage durability and extended use time. The hydrogel bilayers realize the walking of the hydrogel and soft gripper through the change in water temperature, which has potential applications in soft actuators, artificial muscles and soft robots.

## 2. Materials and Methods

### 2.1. Materials

Chitosan (CS), with the deacetylation degree of above 95%, viscosity 100–200 mPa·s, and Montmorillonite (MMT), were purchased from the Macklin Reagent Company (Shanghai, China). Agar, with the relative molecular weight of 3000–9000 Da, was purchased from the Solarbio Reagent Company (Beijing, China). N-isopropylacrylamide (NIPAM) and acrylamide (AM) were obtained from the Aladdin Reagent Company (Shanghai, China). All chemical reagents were of analytical grade, and all solvents were deionized water.

### 2.2. Preparation of Smart Hydrogel Bilayers

**Figure 1:**
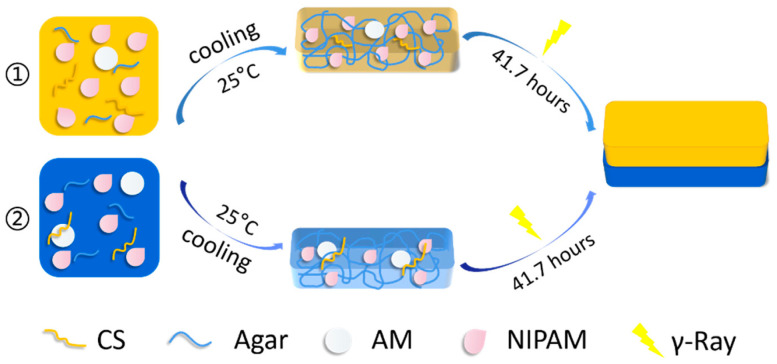
Schematic illustration of the manufacturing process of the temperature-driven hydrogel bilayer actuator.

The preparation processes of CS/agar/P(NIPAM-co-AM) smart hydrogel bilayers are shown in Figure 1. The hydrogel bilayers are composed of two layers, and both layers are prepared by γ ray irradiation. Firstly, chitosan powders were dispersed into the 1 v/v% acetic acid aqueous solution via rapid stirring and stirred for 20 min until they were dissolved. Then, agar powders were dispersed in deionized water at 95 °C to form agar solution with a content of 1.5 wt%. NIPAM and AM, with weight ratios of 95:5 and 65:35, respectively, were added into the agar solution via magnetic stirring. The chitosan solution was added into the above solution and mixed well. The resulting solution was poured into cuboid plastic molds and was cooled to room temperature. Then, they were irradiated by using the ^60^Co gamma ray (dose rate: 0.48 kGy·h^−1^). They were placed together in the oven to glue the two layers together. The top hydrogel layer (C_1_A_1.5_N_9.5_A_0.5_) was fabricated with 9.5 wt% of NIPAM and 0.5 wt% of AM. The bottom hydrogel layer (C_1_A_1.5_N_6.5_A_3.5_) was fabricated with 6.5 wt% of NIPAM and 3.5 wt% of AM. In order to improve mechanical properties, the temperature-sensitive layer with CS/agar/MMT/NIPAM (C_1_A_1.5_M_1_N_9.5_) hydrogel and the non-temperature-sensitive layer with C_1_A_1.5_N_6.5_A_3.5_ hydrogel were also prepared. The preparation method was as follows: MMT was dispersed in water, then agar powders were dispersed at 95 °C until dissolved; NIPAM and CS solution were added to the above solution via magnetic stirring to form the solution with 1 wt% MMT, 1.5 wt% agar, 9.5 wt% NIPAM and 1 wt% CS. Then, the CS/agar/MMT/PNIPAM layer was irradiated by using ^60^Co radiation source (dose rate: 0.48 kGy·h^−1^).

### 2.3. Microstructure and Morphology Characterization

Fourier transform infrared spectroscopy spectra of the hydrogels were measured by a Bruker OPUS 80 V spectrometer (Bruker, Germany) in the range of 4000–500 cm^−1^. The hydrogel bilayers were frozen and dried in a vacuum freeze dryer. The cross-section of this prepared hydrogel was sputtered with gold for observation by scanning electron microscopy (Hitachi S4800, Tokyo, Japan).

### 2.4. Swelling Test

The prepared CS/agar/P(NIPAM-co-AM) hydrogels were dried in a vacuum drying oven until the constant weight was obtained. Then, the dry hydrogels were immersed in deionized water at room temperature. After drying the surface water immediately with filter paper, the swollen samples were weighed again at regular time intervals. This process was repeated until the samples were constant in weight. Swelling rate (SR) was measured according to a previously reported method [32]:

(1) $$SR(\%)=(\frac{W_{t}-W_{d}}{W_{d}})\times 100$$

where *W_d_* is the initial weight of the dry sample and *W_t_* is the weight of the sample after swelling.

### 2.5. Tensile Property Test

The mechanical properties of temperature-sensitive hydrogels were tested at room temperature by using a desktop computerized tension tester (HZ-1004B, Lixian Inc., Guangzhou, China). When measuring tensile properties, the hydrogels were cut into dumbbell-shaped samples (1–2 mm thickness). Each set of test samples was at least 3 parallel samples.

### 2.6. Antibacterial Test

The solidified medium was heated in a microwave oven until the medium was cooled to approximately 40–50 °C, then the above-mentioned spare bacterial solution was added to the culture medium at a concentration of 10^6^ CFU·mL^−1^. The solution was then mixed and poured into a disposable Petri dish (diameter 90 mm). The Petri dish contained 15 mL of culture medium. The sterilized sample (diameter 8 mm) was tested on the corresponding solid medium with an electric heating incubator at 37 °C for 18 h. Then, the size of the inhibition zone was observed and measured.

### 2.7. Temperature Response Test

The hydrogels were immersed in water at 25 °C until they were constant in weight. Then, these samples were immersed in water at 30 °C. After soaking for 12 h, the hydrogels were added to water at 35, 40, 45, 50, 55 or 60 °C and soaked for 12 h. The temperature swelling was determined based on the following equation [8]:

(2) $$Q_{t}=\frac{W_{t}}{W_{i}}$$

where *W_i_* is the initial weight of hydrogel at 25 °C and *W_t_* is the weight of the sample after heating.

## 3. Results and Discussion

### 3.1. Construction and Characterization of Bilayered Hydrogel

The CS/agar/P(NIPAM-co-AM) composite hydrogels with different AM content were prepared by gamma radiation. The boundary of the hydrogel bilayers in hot water can be observed, and no apparent cracks are observed.

**Figure 2:**
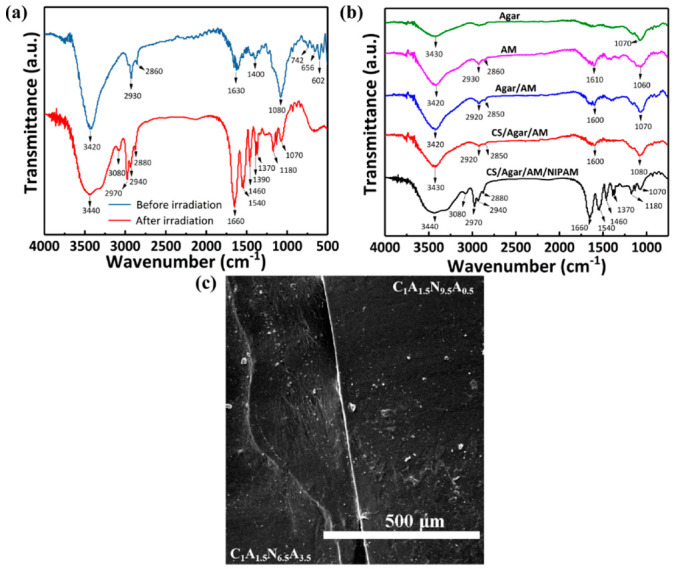
FT-IR spectra and SEM of the hydrogel. (**a**) Comparison of FT-IR spectra of CS/agar/P(NIPAM-co-AM)-gel before and after irradiation. (**b**) Comparison of FT-IR spectra of gels with different systems. (**c**) SEM image of hydrogel bilayers.

The FT-IR spectra of the hydrogels before and after irradiation are shown in Figure 2a. The bands at 742 cm^−1^, 656 cm^−1^, and 1602 cm^−1^ are the RCH=CH_2_ and C=C. After irradiation, these bands disappear. This indicates that the AM and NIPAM were successfully cross-linked by gamma irradiation [33,34]. The band at 2970 cm^−1^ is the stretching vibrations of the -CH(CH_3_)_2_ group. Furthermore, the bands at 1370 cm^−1^, 1390 cm^−1^ and 1540 cm^−1^ correspond to the -CH(CH_3_)_2_ and secondary amides groups in the structure of PNIPAM. In combination with Figure 2b, the bands at 1180 cm^−1^ and 1070 cm^−1^ correspond to the C-O stretching vibrations in the structure of agar. By comparing the IR spectra of several hydrogels in Figure 2b, the -OH and C-O bands are slightly offset. This indicates that the hydrogel contains chitosan. In summary, the composite hydrogel was successfully prepared. The scanning electron microscope scan of the cross-section of the hydrogel bilayers is shown in Figure 2c. In the SEM image, the interface between the two layers can be seen, and the two layers of gel are bonding together. The prepared two layers of hydrogels were closely bonded together and any air between them was squeezed out as far as possible. Then, the two layers of hydrogels were bonded together via a physical cross-link and functional group. The clear interface is probably due to the different densities of the two bilayers.

### 3.2. The Properties of CS/Agar/P(NIPAM-co-AM) Hydrogel

#### 3.2.1. The Swelling Property of CS/Agar/P(NIPAM-co-AM) Hydrogel

**Figure 3:**
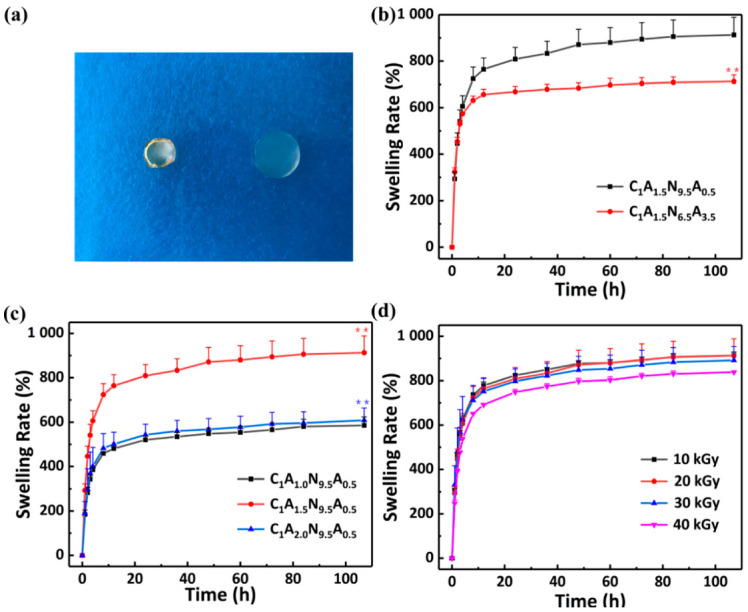
The swelling properties of the CS/agar/P(NIPAM-co-AM) hydrogel. (**a**) Optical photographs of dry hydrogel and hydrogel in equilibrium swelling; the swelling rate (%) of the CS/agar/P(NIPAM-co-AM) hydrogel prepared with (**b**) different ratios of NIPAM and AM, (**c**) different contents of agar and (**d**) different irradiation doses. An asterisk indicates statistically significant differences (** *p* < 0.005; *n* = 3) compared to the previous samples at equilibrium swelling.

The swelling capacity of hydrogel is important for the internal structure analysis and the driving of the hydrogel bilayers. The curves of the swelling rate of CS/agar/P(NIPAM-co-AM) hydrogels with time are shown in Figure 3. When the swelling reached equilibrium, the cross-section of the hydrogel is four times that of the dry gel. As the molecules with hydrophilic groups have a high affinity for water, they attract water molecules easily. The hydrogel can quickly absorb water in a short time due to the capillary action of the pore size of the hydrogel. As the time increased, the growth rate of the swelling rate gradually slows down, and reaches the saturation (equilibrium swelling) in 107 h. The respective swelling rates of the two layers of the hydrogel were measured (Figure 3a). The swelling capability of C_1_A_1.5_N_6.5_A_3.5_ hydrogel is lower than that of C_1_A_1.5_N_9.5_A_0.5_ hydrogel, indicating an increased cross-linking site of AM and an enhanced degree of cross-linking.

In addition, Figure 3c illustrates the swelling rate curves of hydrogels with different agar concentrations. For the hydrogels with 1 wt% agar, the swelling ratio is 586 ± 39%. For hydrogel with 1.5 wt% agar, its swelling ratio displays a maximum of 913 ± 76%. With the increase in agar content, more hydrophilic groups exist in the hydrogel, which improves the ability of hydrogels to attract water molecules. The swelling rate of CS/agar/P(NIPAM-co-AM) hydrogel decreases significantly as the agar content increases to 2 wt%. This indicates that, due to the continuous increase in agar, the cross-linking degree of hydrogel is increased, and the pore becomes smaller, which reduces the water carrying capacity of the hydrogel. Here, the change in swelling rates at various doses are shown in Figure 3d. As the irradiation dose increases, the swelling ratio consequently decreases. The increase in the cross-linking degree is attributed to the distance decrease of the cross-linking point; it can accommodate fewer solvent molecules and results in a lower swelling rate [35]. As the hydrogel with a dose of less than 20 kGy has a lower degree of cross-linking, the sparse three-dimensional network is more beneficial for the entry of water. In summary, the parameters of the 1.5 wt% agar and 20 kGy irradiation dose are selected for subsequent experiments according to the swelling rate. In addition, significant differences in hydrogel swelling rates are compared sequentially. A significant difference in Figure 3b is C_1_A_1.5_N_6.5_A_3.5_ hydrogel compared to C_1_A_1.5_N_9.5_A_0.5_ hydrogel. Significant differences in Figure 3c are C_1_A_1.5_N_9.5_A_0.5_ hydrogel compared to C_1_A_1.0_N_9.5_A_0.5_ hydrogel and C_1_A_2.0_N_9.5_A_0.5_ hydrogel compared to C_1_A_1.5_N_9.5_A_0.5_ hydrogel.

#### 3.2.2. Mechanical Properties

**Figure 4:**
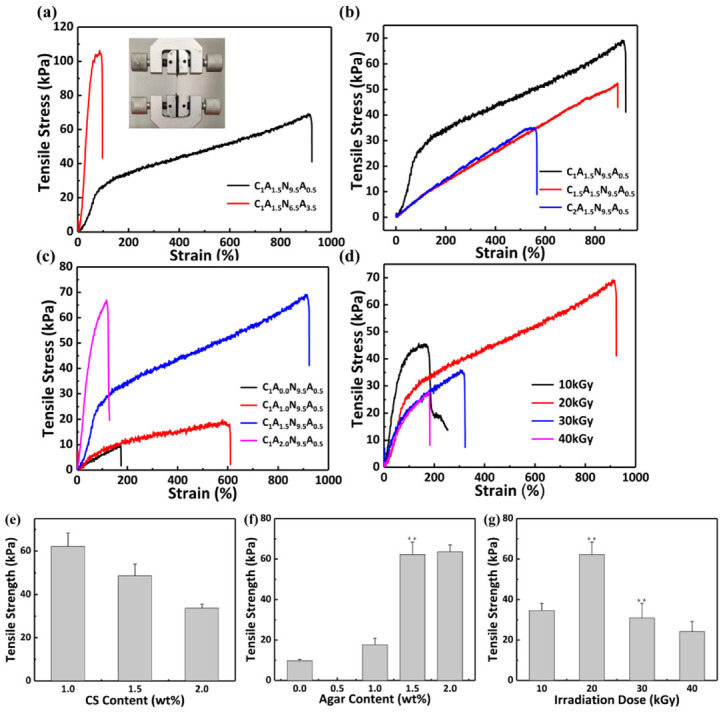
The tensile property of the CS/agar/P(NIPAM-co-AM) hydrogel. The tensile stress (kPa) of the CS/agar/P(NIPAM-co-AM) hydrogel with (**a**) different ratios of NIPAM:AM; (**b**) different contents of CS; (**c**) different contents of agar; (**d**) different irradiation doses. Tensile strength with (**e**) different contents of CS; (**f**) different contents of agar; (**g**) different irradiation doses. An asterisk indicates statistically significant differences (** *p* < 0.005).

The tensile stress-strain curves of the CS/agar/P(NIPAM-co-AM) hydrogel bilayers are shown in Figure 4a. Mechanical properties are enhanced with the increase in acrylamide due to the increased degree of cross-linking of the hydrogel.

The effects of CS and agar on the mechanical properties of the whole system were investigated by changing the content of CS and agar. Figure 4b shows that the tensile stress is gradually reduced as the CS content increases, which is due to the degradation of chitosan under irradiation and the uniformity of CS solution. The mechanical properties of the hydrogel are optimal when the content of chitosan is 1 wt%. As shown in Figure 4c, the strain and the tensile stress are gradually enhanced during the increase in agar content. The maximum tensile stress can reach 69 kPa when the agar content of hydrogel is 1.5 wt%, which is approximately seven times as much as that of the non-agar hydrogel. Furthermore, the tensile strain can reach 913%. The tensile strength of the hydrogels with agar can be enhanced in comparison to non-agar hydrogels [36]. Prior to irradiation, the addition of agar causes the hydrogel to form a hard and brittle first network. After cross-linking by irradiation, P(NIPAM-co-AM) and chitosan can form a soft and tough network. The interaction of networks improves the mechanical properties. When the content of agar is increased to 2 wt%, the tensile strength of the hydrogel is decreased. The tensile stress is similar to the C_1_A_1.5_N_9.5_A_0.5_ hydrogel, but the strain is only 118%. The cross-linking degree is enhanced when too much agar is added, making the hydrogel brittle [31]. Figure 4d shows the variation of the tensile property of hydrogel with the doses. When the irradiation dose is not more than 20 kGy, the tensile strength is increased with the increase in irradiation dose. Irradiation can induce more free radicals, which make more polymer molecular chains cross-linked together and enhance the cross-linking degree of the hydrogel [37,38]. However, when irradiation dose is continued to increase, the degree of cross-linking is increased, which leads to smaller pore size inside the hydrogel and lower tensile strength [39]. The degradation of chitosan is enhanced during irradiation and leads to a lower mechanical property [35]. The CS/agar/P(NIPAM-co-AM) hydrogel shows the best tensile performance when the mass fraction of CS and agar are 1 and 1.5, respectively, and the irradiation dose is 20 kGy, with a tensile strength of 69 kPa and a strain of 913%. Significant differences in tensile strength of hydrogels are compared sequentially. Significant differences in Figure 4e are C_1.5_A_1.5_N_9.5_A_0.5_ hydrogel compared to C_1_A_1.5_N_9.5_A_0.5_ hydrogel and C_2.0_A_1.5_N_9.5_A_0.5_ hydrogel compared to C_1.5_A_1.5_N_9.5_A_0.5_ hydrogel. Significant differences in Figure 4f are C_1_A_1_N_9.5_A_0.5_ hydrogel compared to C_1_A_0_N_9.5_A_0.5_ hydrogel, C_1_A_1.5_N_9.5_A_0.5_ hydrogel compared to C_1_A_1_N_9.5_A_0.5_ hydrogel and C_1_A_2.0_N_9.5_A_0.5_ hydrogel compared to C_1_A_1.5_N_9.5_A_0.5_ hydrogel. Significant differences in Figure 4g are hydrogel (20 kGy) compared to hydrogel (10 kGy), hydrogel (30 kGy) compared to hydrogel (20 kGy) and hydrogel (40 kGy) compared to hydrogel (30 kGy).

#### 3.2.3. Antibacterial Test

**Figure 5:**
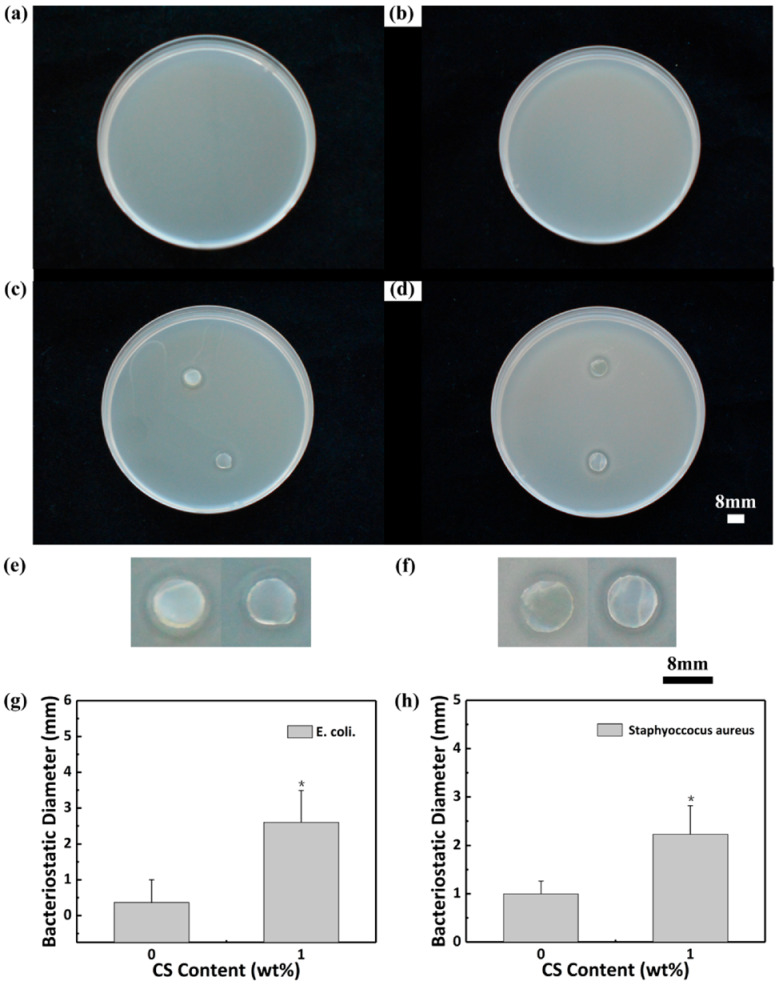
Antibacterial test photographs. The control groups of (**a**) *E. coli* and (**b**) *Staphylococcus aureus*. Bacteriostasis situation of (**c**) *E. coli* and (**d**) *Staphylococcus aureus* (top: hydrogel with CS, bottom: hydrogel without CS). Partially enlarged view of (**e**) and (**f**) (left: hydrogel with CS, right: hydrogel without CS; the diameter of hydrogel samples is 8 mm.). Bacteriostatic diameter of (**g**) *E. coli* and (**h**) *Staphylococcus aureus*. An asterisk indicates statistically significant differences (* *p* < 0.05).

The antimicrobial activity of these two hydrogels was measured with *E. coli* and *Staphylococcus aureus*. Figure 5 shows the antibacterial experimental results of CS/agar/P(NIPAM-co-AM) and agar/P(NIPAM-co-AM) hydrogels. The control groups of *E. coli* and *Staphylococcus aureus* are shown in Figure 5a and Figure 5b, respectively. *E. coli* bacteria colonies do not appear on either the CS/agar/P(NIPAM-co-AM) hydrogel and the agar/P(NIPAM-co-AM) hydrogel (Figure 5c,e). The inhibition zone diameter of the 1 wt% chitosan hydrogel is approximately 3.3 mm, and that of the non-chitosan hydrogel was 1.1 mm. CS/agar/P(NIPAM-co-AM) hydrogel and agar/P(NIPAM-co-AM) hydrogel do not appear on *Staphylococcus aureus* colonies. The antibacterial diameter of the hydrogel with 1 wt% chitosan is 2.9 mm, and that of the non-chitosan hydrogel is 1.1 mm (Figure 5d,f). This indicates that the antibacterial performance of the C_1_A_1.5_N_9.5_A_0.5_ is better than that of the C_0_A_1.5_N_9.5_A_0.5_ [40]. The antibacterial properties of the C_1_A_1.5_N_9.5_A_0.5_ hydrogel are better than the cellulose/chitosan composite hydrogel [41]. The column graphs (Figure 5g,h) show the diameter of the inhibition zone of *E. coli* and *Staphylococcus aureus*, respectively. Significant differences in inhibition zone diameter of the C_1_A_1.5_N_9.5_A_0.5_ are compared to the C_0_A_1.5_N_9.5_A_0.5_.

### 3.3. Temperature Sensitivity and Temperature-Driven Deformation

#### 3.3.1. The VPTT of the Hydrogel

**Figure 6:**
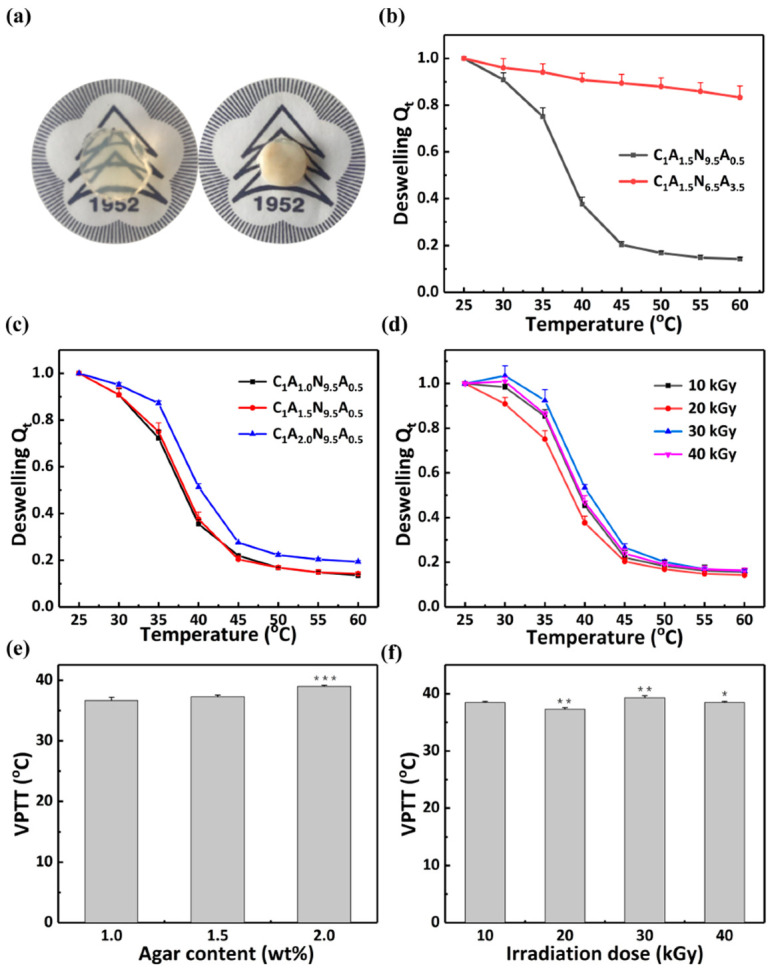
Temperature responsiveness. (**a**) The state of the hydrogel with the critical temperature. Deswelling of (**b**) the C_1_A_1.5_N_9.5_A_0.5_ and C_1_A_1.5_N_6.5_A_3.5_ hydrogels with increasing temperature, (**c**) different content of agar with increasing temperature and (**d**) the C_1_A_1.5_N_9.5_A_0.5_ hydrogels of different doses with increasing temperature. VPTT of samples with (**e**) different agar (**f**) different irradiation doses. An asterisk indicates statistically significant differences (* *p* < 0.05, ** *p* < 0.005, *** *p* < 0.001; *n* = 3) as compared with the previous samples.

Volume phase transition temperature is an important indicator with which to measure the temperature sensitivity of hydrogels. The water loss rate of the hydrogels with increasing temperature was measured, and Boltzmann fitting was performed on the experimental data [42,43]. The volume phase transition temperature (VPTT) [44,45] of the hydrogel was determined as the inflection point of the curve. When the temperature reaches VPTT, the color of the hydrogel instantly becomes white and the volume is decreased over a short time (Figure 6a). The VPTT of two hydrogel layers can be adjusted by changing the AM content. The VTPP of the C_1_A_1.5_N_9.5_A_0.5_ hydrogel is 37.3 ± 0.3 °C. Due to the increase in AM content (Figure 6b), the VPTT of C_1_A_1.5_N_6.5_A_3.5_ hydrogel cannot be reached. At a specific temperature (37.3–60 °C), the volume of the two layers of hydrogel can change to different degrees, which will lead to the bending of the hydrogel bilayers [27,33].

In addition, the temperature sensitivities of the composite hydrogel under different content of agar and different irradiation doses were tested. From 25 °C to 60 °C, Q_t_ of hydrogel decreases rapidly (Figure 6c,d). At temperatures above the VPTT, it is in a hydrophobic state. This behavior can be due to the thermal dissociation of hydrating water from the polymer chains and the hydrogen bonding weakening [46,47]. In Figure 6c, the VPTT of the C_1_A_1_N_9.5_A_0.5_ hydrogel, the C_1_A_1.5_N_9.5_A_0.5_ hydrogel and the C_1_A_2_N_9.5_A_0.5_ hydrogel is 36.7 ± 0.5 °C, 37.3 ± 0.3 °C and 39.0 ± 0.2 °C, respectively. Therefore, the VPTT of hydrogels increases with the increase in agar content. The VPTT of the hydrogel with 2 wt% agar changes more significantly. Hydrophilic substances such as acid [48] and ethylene glycol [49] can result in a high VPTT of PNIPAM composite hydrogel. Above the VPTT, the hydrophobic groups (isopropyl -CH(CH_3_)_2_) in NIPAM play dominant role in the hydrogel. The increase in the specific gravity of the hydrophilic molecules can improve the water absorption capability [50,51,52]. The temperature sensitivities of hydrogels were measured to explore the effect with irradiation doses (Figure 6d). The VPTT of the hydrogels which were prepared under the radiation dose of 10 kGy, 20 kGy, 30 kGy and 40 kGy are 38.5 ± 0.2 °C, 37.3 ± 0.3 °C, 39.3 ± 0.4 °C and 38.5 ± 0.2 °C respectively. The irradiation dose does not affect the ratio of hydrophilic and hydrophobic groups in the hydrogel. Hence, hydrogels polymerized with different doses show no linear relationship between irradiation dose and the VPTT. Significant differences in Figure 6e are C_1_A_1.5_N_9.5_A_0.5_ hydrogel compared to C_1_A_1.0_N_9.5_A_0.5_ hydrogel and C_1_A_2.0_N_9.5_A_0.5_ hydrogel compared to C_1_A_1.5_N_9.5_A_0.5_ hydrogel. Significant difference in Figure 6f are hydrogel (20 kGy) compared to hydrogel (10 kGy), hydrogel (30 kGy) compared to hydrogel (20 kGy) and hydrogel (40 kGy) compared to hydrogel (30 kGy).

#### 3.3.2. Temperature Responsive Behaviors of the Hydrogel Bilayers

**Figure 7:**
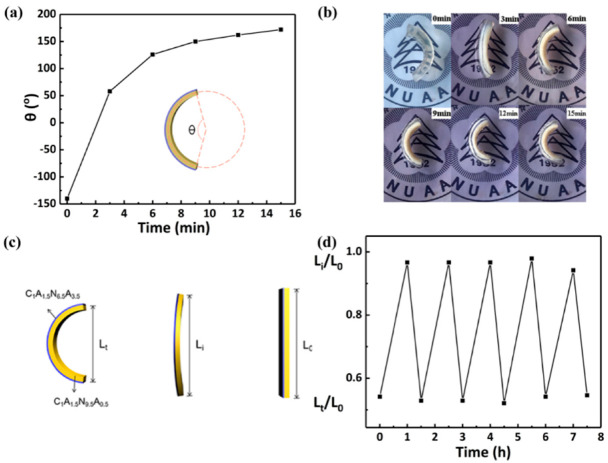
Bending of hydrogel bilayers. (**a**) The curve and (**b**) the photo of the central angle of the hydrogel bilayers in the first 15 min. (**c**) The schematic diagram of hydrogel bilayer and (**d**) the repeated reversible behavior of hydrogels.

The responsiveness and reversibility of hydrogel bilayers are essential for their practical application in actuators. Therefore, hydrogel bilayers with the thicknesses of 3 mm C_1_A_1.5_N_9.5_A_0.5_ and 1 mm C_1_A_1.5_N_6.5_A_3.5_ in the swelling equilibrium state were fabricated to investigate their responsiveness and reversibility. Figure 7a,b show the change in the central angle of hydrogel in hot water. After 15 min, its central angle changes by 312 degrees. Further, L_t_/L_0_ and L_i_/L_0_ are used to quantify the temperature response of the hydrogel, where L_t_ and L_i_ are the distance between the two endpoints of the hydrogel bilayers in hot water and at room temperature, respectively, and L_0_ is the total length of the hydrogel bilayer (Figure 7c). As shown in Figure 7d, the response of the hydrogel can maintain at least 94% of the original L_t_/L_0_ and L_i_/L_0_ after five cycles. Therefore, the as prepared hydrogel bilayers show excellent reversibility.

#### 3.3.3. Thermo-Driven Move and Thermo-Deformation of Hydrogel Bilayers

**Figure 8:**
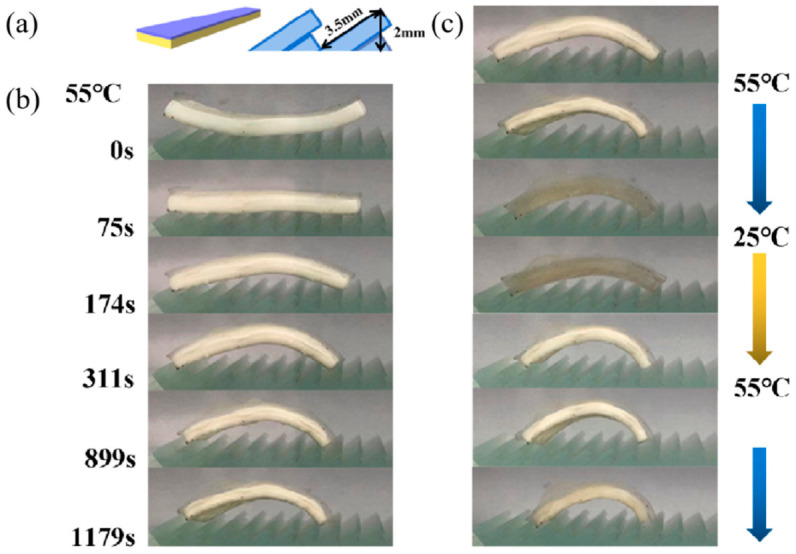
Thermo-driven move of hydrogel bilayers. (**a**) The schematic diagrams of the hydrogel bilayers the shape of the glass. (**b**) Photos of the bilayers self-moving in hot water. (**c**) Photos of the bilayers actuated by heating and cooling of the water.

In order to realize jumping forward in the temperature-controlled water, composite hydrogel bilayers with a temperature-sensitive bending response were designed. As shown in Figure 8a, their top view is an equilateral trapezoid with a short side of 0.45 cm, a long side of 1 cm and a height of 3.2 cm. The thickness of the C_1_A_1.5_N_9.5_A_0.5_ layer is 0.3 cm and the C_1_A_1.5_N_6.5_A_3.5_ layer is 0.1 cm. The front and back widths of the hydrogel bilayers are different. The hydrogel bilayers are placed on a jagged glass block. The hydrogel bilayers can move spontaneously with the change in temperature.

The hydrogel bilayers are significantly bent in 55 °C hot water (Figure 8b) and their rear foot moves 4.9 mm forward. The elastic potential energy is transformed into kinetic energy, which causes the hydrogel to move forward. Figure 8c and the video in Supplementary Material S1 show the reversible processes of the hydrogel bilayers bending, and the hydrogel can move 8.4 mm forward under this circumstance. The hydrogel actuator will continuously move forward through the control of temperature.

**Figure 9:**
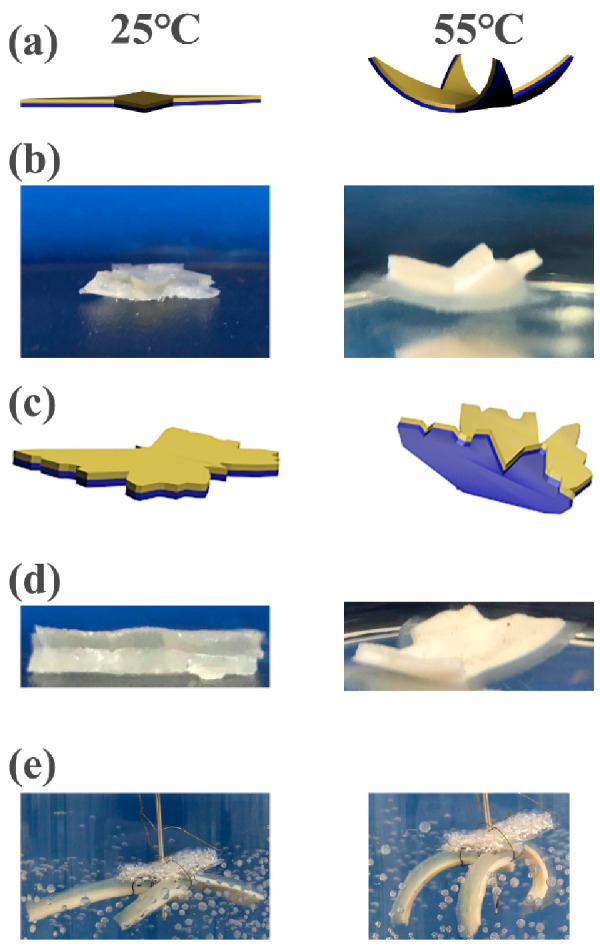
The schematic diagrams and photos of the hydrogel at different temperatures. (**a**) The schematic diagrams and (**b**) the photos of the hydrogel bilayers of “flower”. (**c**) The schematic diagrams and (**d**) the photos of the hydrogel bilayers of “butterfly”. (**e**) The soft gripper of the hydrogel bilayers of the hot water.

The hydrogel bilayers in the shape of a flower were designed to imitate the movement of petals (Figure 9a,b). The “petals” can bloom horizontally at room temperature, while they converge inwards in hot water. Additionally, the hydrogel bilayers were cut into the shape of a butterfly to imitate the spreading wings (Figure 9c,d). The wings can be stimulated to erect in hot water. In addition, three strips of hydrogels bilayers were fixed together with copper wires and placed in hot water at 55 °C (Figure 9e). The hydrogel bilayers bend quickly to realize the grasping action. The thicknesses of the C_1_A_1.5_N_9.5_A_0.5_ and C_1_A_1.5_N_6.5_A_3.5_ layers are 1.9 mm and 2.2 mm, respectively.

### 3.4. CS/Agar/MMT/PNIPAM Hydrogel

**Figure 10:**
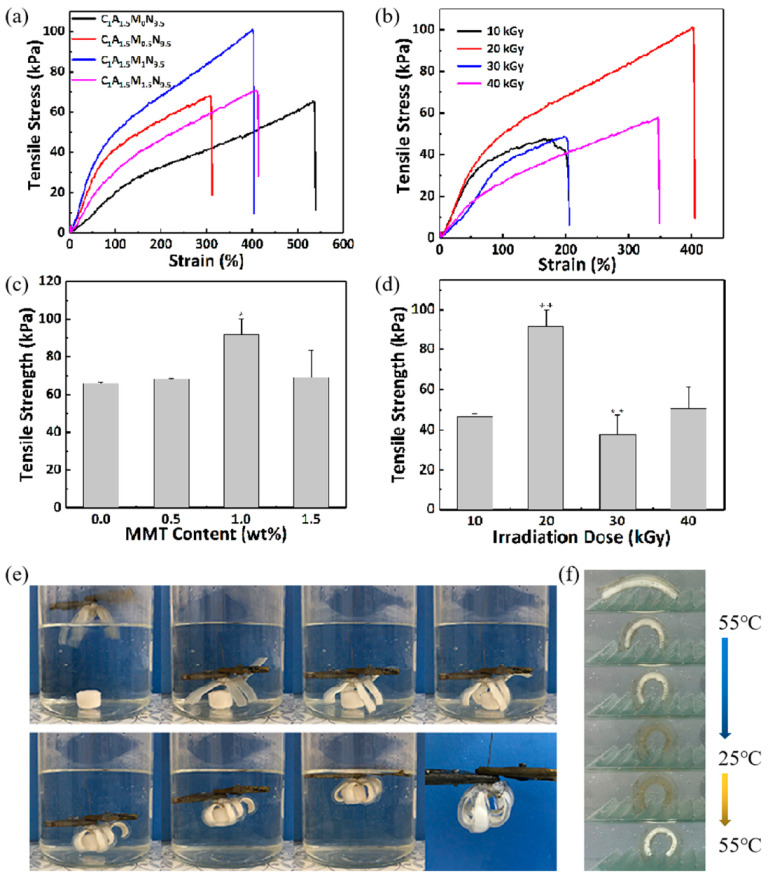
The property and the application of CS/agar/MMT/PNIPAM hydrogel. Tensile stress-strain with (**a**) different content of MMT and (**b**) different irradiation dose. Tensile strength with (**c**) different content of MMT, (**d**) different irradiation dose. An asterisk indicates statistically significant differences (* *p* < 0.05, ** *p* < 0.005). (**e**) The soft gripper of the hydrogel bilayers in the hot water. (**f**) Photos of the bilayer actuated by heating and cooling of the water.

CS/agar/P(NIPAM-co-AM) and CS/agar/MMT/PNIPAM hydrogel bilayers were also prepared. The tensile stress of the hydrogel without MMT is 65 kPa. The tensile stress reaches 101 kPa when 1 wt% MMT is added (Figure 10a). The MMT acts as a cross-linking agent in the hydrogel system, which increases the cross-linking point and degree of the hydrogel. However, the tensile stress decreases to 71 kPa when the content of MMT increases to 1.5 wt%. The high content of MMT leads to the agglomeration of hydrogels. The hydrogel prepared by 20 kGy irradiation shows the best tensile mechanical property (Figure 10b). Significant differences in Figure 10c are C_1_A_1.5_M_0.5_N_9.5_ hydrogel compared to C_1_A_1.5_M_0_N_9.5_ hydrogel, C_1_A_1.5_M_1_N_9.5_ hydrogel compared to C_1_A_1.5_M_0.5_N_9.5_ hydrogel and C_1_A_1.5_M_1.5_N_9.5_ hydrogel compared to C_1_A_1.5_M_1_N_9.5_ hydrogel. Significant differences in Figure 10d are hydrogel (20 kGy) compared to hydrogel (10 kGy), hydrogel (30 kGy) compared to hydrogel (20 kGy) and hydrogel (40 kGy) compared to hydrogel (30 kGy).

The hydrogel bilayers are employed to make temperature-sensitive soft grippers. A cylindrical hydrogel is placed at the bottom. As shown in Figure 10e and the video in Supplementary Material S2, the “arms” gradually coil around the object as the temperature changes. Then the copper wire can be hoisted up to realize the underwater grasping operation. Figure 10f shows the self-moving experiment of hydrogel bilayers under temperature control, with a movement of 7.2 mm. The thickness of the C_1_A_1.5_N_6.5_A_3.5_ layer is 1 mm and the C_1_A_1.5_M_1_N_9.5_ layer is 3 mm.

## 4. Conclusions

In this work, CS/agar/P(NIPAM-co-AM) and CS/agar/MMT /PNIPAM hydrogel bilayers were prepared via irradiation and used as a temperature-sensitive actuator. The hydrogel bilayers can bend as the water warmed up and return to their original state after cooling. They show excellent reversibility during cyclic reversible bending. They can bend under temperature stimulation in hot water, which can realize self-moving as an actuator and realize a grasping operation as a soft gripper. They show good antibacterial property, which may extend their service life. These temperature-sensitive hydrogel bilayers have potential applications in soft robots, soft gripper, artificial muscles, etc.

## Data Availability

The data presented in this study are available on request from the corresponding author.

## Supplementary Materials

The following are available online at https://www.mdpi.com/article/10.3390/polym13111753/s1, Video S1: Thermo-driven move of hydrogel bilayers; Video S2: The hydrogel bilayers used as soft gripper.
